# Evolution in a Community Context: towards Understanding the Causes and Consequences of Adaptive Evolution in the Human Gut Microbiota over Short Time Scales

**DOI:** 10.1128/mSystems.00832-21

**Published:** 2021-08-24

**Authors:** Pauline Deirdre Scanlan

**Affiliations:** a APC Microbiome Ireland and School of Microbiology, University College Cork, Cork, Ireland

**Keywords:** coevolution, gut microbiota, microbial evolution, microbiome, microcosm, bacteriophages, microbial ecology

## Abstract

How important is adaptive evolution to the unique diversity that we can observe for each individual human gut microbiome? How do gut microbes evolve in response to changes in their environment, and how does evolution in real time impact microbial functionality in the context of host health? My interdisciplinary research uses *in vitro* microcosm models to test how different abiotic and biotic factors impact microbial evolution in a community context. We complement this approach by tracking focal species as they evolve in real time and in their natural environment of the human gut. Our aim is to provide a better understanding of how the dynamics and outcomes of microbial evolution differ between individual gut environments, and in response to different selection pressures, so that we can move closer to rational gut microbiome treatments that promote host health and prevent and treat human disease.

## COMMENTARY

## TOWARDS LINKING ADAPTIVE EVOLUTION TO PHENOTYPIC VARIATION IN THE HUMAN GUT MICROBIOTA

Understanding how the gut microbiota affects the human host phenotype has become a central research focus across a broad span of scientific disciplines ranging from microbiology, medicine, immunology, nutrition, and physiology to neuroscience (1–3). Yet, despite extensive research efforts and considerable investment we still lack a fundamental understanding of how variation in microbial diversity translates to microbial function and ultimately human health and wellbeing (4). There are several reasons for this, with one of the possible explanations being that the current state of the art conceptualizes and studies members of this complex community from a largely static perspective, with microbes, such as different strains and species of bacteria, treated as fixed functional entities. However, this assumption is fundamentally flawed and thankfully is beginning to change due to emerging studies showcasing the capacity for microbes to rapidly evolve over short time scales on and in different body sites including the human gut (5, 6). Crucially, these studies support the idea that the phenotypes and functionality of bacteria have the capacity to change over short periods of time and that microbial evolution in real time has implications for host health (7, 8).

In my lab, we study the adaptive evolution of the human gut microbiota over short time scales (weeks, months, and years). I am interested in understanding how different biotic (bacteriophages and disease onset) and abiotic (resources and xenobiotics) factors shape the evolution and genetic diversity of microbial populations and how genetic changes observed *in vitro*, *in vivo*, and *in silico* ultimately translate to phenotypic and functional diversity in the human gut. To do so, we use *in vitro* microcosm models, together with longitudinally sampling and isolating focal species of interest from the human gut to track evolution as it occurs in its natural environment. We then complement these experiments and studies with further genetic and phenotypic analyses of isolates of interest.

## BACTERIA-PHAGE COEVOLUTION USING *IN VITRO* MICROCOSMS AND THE STUDY OF MICROBIAL POPULATIONS IN NATURE

**FIG 1:**
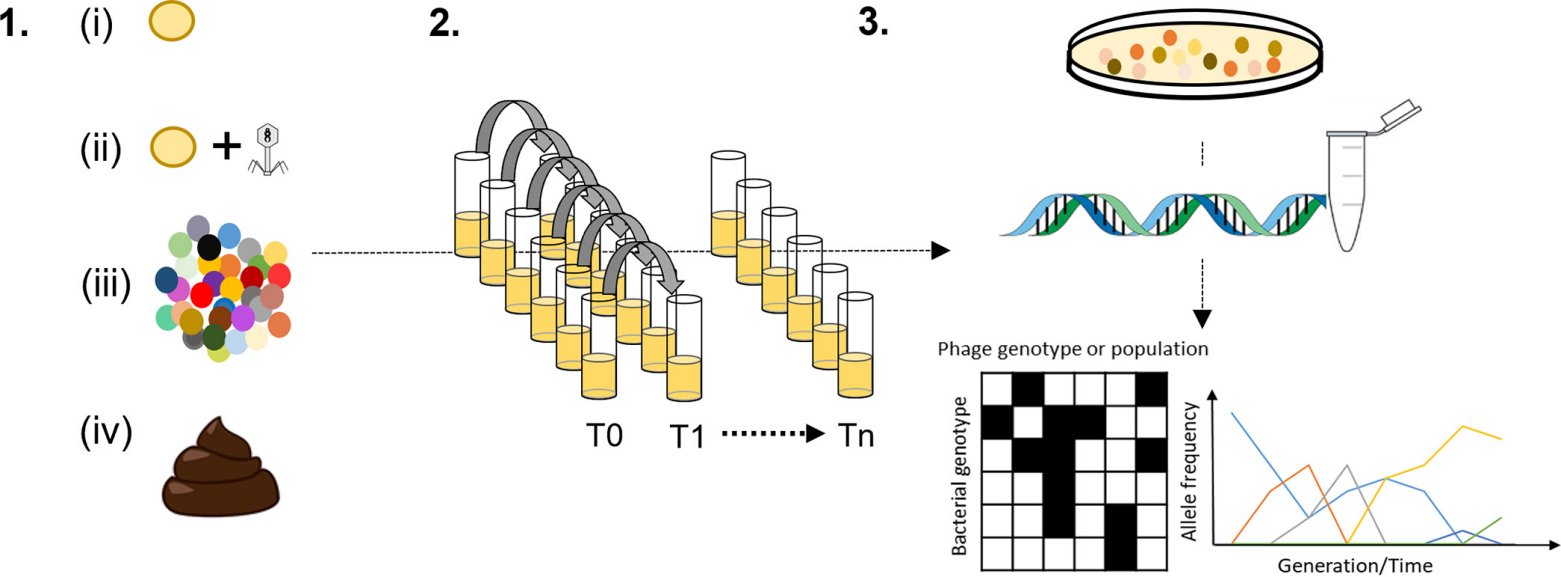
Using *in vitro* microcosms to study the ecology and evolution of the human gut microbiota. 1. Choice of inoculum: (i) single focal species, (ii) focal species plus bacteriophage, (iii) defined community of microbes, (iv) fecal sample(s). 2. *In vitro* model system: batch culture serial transfer—choose number of replicates, time scale of experiments, and variables to manipulate, e.g., resources, xenobiotics, bile salts, etc. 3. Sampling and analysis: temporally sample your model to isolate individual and/or different species of interest for genetic and phenotypic analysis as appropriate to research question/hypothesis. Whole communities can also be sampled and analyzed using 16S rRNA analysis, q-PCR, and/or shotgun metagenomic analysis.

It is predicted that coevolution between phages and their bacterial hosts plays a fundamental role in driving and maintaining the unique genetic diversity we observe in each individual human gut microbiome (9, 10). Nonetheless, our understanding of bacteria and phage coevolutionary dynamics and its potential functional consequences in complex environments including the human gut is greatly limited (9, 11). One powerful approach to address this gap in our knowledge is experimental evolution which enables the study of evolutionary processes and outcomes over different time scales, and in response to different environmental contexts and selection pressures (12, 13) (Fig. 1). Using this reductionist framework, microbes of interest are typically inoculated into vials or plates containing nutrients to support growth and propagated through time using batch culture serial transfer. One can manipulate any number of different abiotic and biotic variables as required, and stocks from different time points can be prepared and stored for later analysis. Experiments can be replicated at scale, and to further facilitate experimentation and data interpretation, model organisms, which are tractable and characterized in detail, are typically used.

In a recent study using the Escherichia coli and PP01 model of bacteria-phage coevolution, we have shown that the simple addition of one environmentally relevant factor present in the human gut altered the dynamics of bacteria-phage coevolution (14). Here, the presence of bile salts dampened the signal for directional selection on phage and host infectivity and resistance ranges, respectively. One explanation, in part, for this effect relates to the negative impact of bile salts on both host and phage population sizes. This is turn has the potential to reduce encounter rates and thus lessen the benefits of generalism in host and parasite resistance and infectivity ranges that are typically observed for host-parasite arms race dynamics. Moreover, we found that bile salts negatively impacted phage viability, as well as phage adsorption efficiency, suggesting that the negative effect of bile salts on phage population sizes may also be partially independent of host population size (14).

These findings clearly exemplify the importance of incorporating more realistic environmental variables into experimental designs. However, to move forward, we need further development of *in vitro* model approaches to better understand the mode, tempo, and dynamics of bacteria-phage coevolution in the gut. In particular, we need to incorporate the use of non-model strains of different gut species that have not undergone extensive lab propagation and potential lab adaptation, together with scaling up the biological complexity of *in vitro* models. As an example, we are currently using a number of different sympatric bacteria and phages that have been isolated from the human gut as inoculum in our models of coevolution. We are also manipulating our experimental design and microcosm experiments to include relevant abiotic factors such as spatial structure (15) together with biotic factors such as the addition of a background community that can be plated out to quantify and study different component species. By adapting the traditional single-species–single-phage microcosm model of coevolution to include non-model organisms and a simple characterized background community that is representative of the major phyla found in the human gut microbiota, we hope to better understand how the dynamics, persistence, and outcomes of bacteria-phage coevolution are shaped in more biologically realistic scenarios.

To complement this experimental approach, we are also tracking bacteria-phage interactions in time (within individuals) and in space (between individuals) in the gut by isolating focal bacterial species of interest from human fecal samples and testing them against potential infective phages in sympatry and allopatry. While more technically challenging to conduct and interpret than more simplified and highly replicated microcosm experiments, time-shift experiments of naturally occurring populations of bacteria and phages coupled to measures of phage local adaption are helping provide a window of insight into the mode and tempo of coevolution as it occurs in real time in the human gut. Of note, these experimental approaches have proved very useful in the study of bacteria-phage coevolution in other naturally occurring communities of microbes (16) and will help facilitate increased understanding of the complex dynamics of interspecies interactions and underlying eco-evolutionary processes that shape the unique diversity of each individual gut microbiome (9).

## HOW DOES THE CHEMICAL ENVIRONMENTAL SHAPE THE ECOLOGY AND EVOLUTION OF THE GUT MICROBIOTA?

In addition to researching the effects of naturally occurring biotic selection pressures on the ecology and evolution of the gut microbiota, we are also using *in vitro* microcosm models to investigate how different gut microbes respond to changes in their chemical environment. This is crucially important as millions of humans, on a daily and global scale, are exposed to a multitude of xenobiotics such as medicines, antibiotics, and antimicrobials as well as components of a Western diet (artificial sweeteners, preservatives, colorings) (17). These xenobiotics represent a diverse range of chemical classes, and given their antimicrobial nature and chemical composition, xenobiotic exposure likely has a range of effects on the ecology and evolution of microbial populations including natural selection for resistance evolution and/or novel substrate utilization.

In particular, antibiotics are perhaps the best-known class of chemicals that can impose lethal selection pressures on microbial populations. Nonetheless, even though the potential for antibiotics to affect the ecology and evolution of the human gut microbiota, with myriad implications for host health, is well recognized, we actually know little about the specific modes and tempo of antibiotic resistance evolution in this complex environment (18). Additionally, antibiotics likely have a range of other effects on community composition and function outside direct selection for resistance evolution alone, which remain largely unexplored. This includes potential ecological opportunity owing to access to novel resources and competitive release which could impose divergent selection on resident species (19).

We are currently focused on using fecal samples as inoculum in our microcosm models to investigate how the same community is affected by different antibiotic classes—not just in terms of community composition but also from an evolutionary perspective. We can look at the mode and tempo of resistance evolution and the underlying genetic details of the process including the relative importance of horizontal gene transfer and the potential pleiotropic effects of mutations by using longitudinal sampling and selective plating coupled to whole-genome sequencing, comparative genomics, and phenotypic analysis. This model-based approach also allows us to investigate how other members of the gut microbiota such as fungi, protists, and phages are impacted by different antibiotics.

## DIVERSITY AND PREVALENCE OF MICROBIAL EUKARYA IN THE HUMAN GUT

In addition to our primary focus on microbial evolution, I continue my work on profiling the prevalence and diversity of microbial eukarya associated with the human gut (20). Unfortunately, microbial eukarya, including fungi and the intestinal protist *Blastocystis*, have been greatly overlooked in gut microbiome studies to date, but similar to bacterial and phage fractions of the gut community, microbial eukarya vary in their diversity and prevalence according to diet, urbanization, and age among other factors (21, 22). However, there remain many technical challenges to their study and knowledge gaps as to how they contribute to gut microbial ecology and functionality (18, 23). As such, further development and optimization of methods (24, 25) together with provision of fundamental primary research data on their ecology and evolution in the human gut are necessary to provide a more holistic picture of how individual species of microbial eukarya together with whole communities of microbes are assembled, interact, evolve, and contribute to human health and wellbeing.

## CONCLUDING REMARKS

Research into the human gut microbiota ultimately seeks to understand how individual species, specific microbial taxonomic groups, and whole communities of microbes contribute to host health and disease. The motivation is that this knowledge will then allow us to somehow “manipulate” the microbiota or microbiome in a manner that can promote human health, prevent disease onset, and treat and/or minimize the effect of disease when it occurs. However, if adaptive evolution can drive phenotypic variation in gut microbes over short time scales and divergent selection pressures are operating in the human gut at the level of the individual, then we need to understand the fundamental dynamics of these eco-evolutionary processes as well as the scale of their consequences in order to harness the potential power and promise of the gut microbiota as a mediating factor in human health.
